# Synergistic Effect on Neurodegeneration by N-Truncated Aβ_4−42_ and Pyroglutamate Aβ_3−42_ in a Mouse Model of Alzheimer's Disease

**DOI:** 10.3389/fnagi.2018.00064

**Published:** 2018-03-08

**Authors:** Jose S. Lopez-Noguerola, Nicolai M. E. Giessen, Maximilian Ueberück, Julius N. Meißner, Charlotte E. Pelgrim, Johnathan Adams, Oliver Wirths, Yvonne Bouter, Thomas A. Bayer

**Affiliations:** Division of Molecular Psychiatry, University Medical Center, Georg-August-University, Goettingen, Germany

**Keywords:** Alzheimer's disease, N-truncated Aβ, pyroglutamate Aβ, neuron loss, intraneuronal Aβ, behavior, transgenic mouse models

## Abstract

The N-terminally truncated pyroglutamate Aβ_3−42_ (Aβ_pE3−42_) and Aβ_4−42_ peptides are known to be highly abundant in the brain of Alzheimer's disease (AD) patients. Both peptides show enhanced aggregation and neurotoxicity in comparison to full-length Aβ, suggesting that these amyloid peptides may play an important role in the pathogenesis of AD. The aim of the present work was to study the direct effect of the combination of Aβ_pE3−42_ and Aβ_4−42_ on ongoing AD-related neuron loss, pathology, and neurological deficits in transgenic mice. Bigenic mice were generated by crossing the established TBA42 and Tg4-42 mouse models expressing the N-truncated Aβ peptides Aβ_pE3−42_ and Aβ_4−42_, respectively. After generation of the bigenic mice, detailed phenotypical characterization was performed using either immunostainings to evaluate amyloid pathology or quantification of neuron numbers using design-based stereology. The elevated plus maze was used to study anxiety levels. In order to evaluate sensori-motor deficits, the inverted grid, the balance beam and the string suspension tasks were applied. We could demonstrate that co-expression of Aβ_pE3−42_ and Aβ_4−42_ accelerates neuron loss in the CA1 pyramidal layer of young bigenic mice as seen by reduced neuron numbers in comparison to single transgenic homozygous mice expressing either Aβ_pE3−42_ or Aβ_4−42_. This observation coincides with the robust intraneuronal Aβ accumulation observed in the bigenic mice. In addition, loss of anxiety and motor deficits were enhanced in an age-dependent manner. The sensori-motor deficits correlate with the abundant spinal cord pathology, as demonstrated by robust intracellular Aβ accumulation within motor neurons and extracellular Aβ deposition. Our observations demonstrate that a combination of Aβ_pE3−42_ and Aβ_4−42_ has a stronger effect on ongoing AD pathology than the peptides alone. Therefore, Aβ_pE3−42_ and Aβ_4−42_ might represent excellent potential therapeutic targets and diagnostic markers for AD.

## Introduction

Alzheimer's disease (AD) is the most common type of dementia worldwide. Pathologically, AD represents a progressive neurodegenerative disorder characterized by the accumulation of amyloid-β protein (Aβ), neurofibrillary tangles comprising hyperphosphorylated Tau, and neuronal loss. The amyloid hypothesis regards the accumulation of Aβ in the brain as a fundamental event in the pathogenesis of AD (Hardy and Allsop, 1991). The production of Aβ is the result of the sequential cleavage of the larger amyloid precursor protein (APP) by the β- and γ-secretases (Selkoe, 1998). In addition to full length Aβ_1−40_ and Aβ_1−42_ peptides starting with an aspartate at position 1, a variety of different N-truncated Aβ peptides have been identified in AD brains (reviewed in Bayer and Wirths, 2014). Among these variants a truncated peptide starting at phenylalanine at position 4 (Aβ_4−42_) was reported in the brain of AD and Down's syndrome (DS) patients for the first time by Masters et al. already more than 30 years ago (Masters et al., 1985). Later studies corroborated the presence and relatively abundance of Aβ_4−42_ in aged controls, vascular dementia and AD patients (Lewis et al., 2006). Supporting these findings, Portelius et al. (2010) using immunoprecipitation and mass spectrometry analysis, reported that the dominating Aβ isoforms in the hippocampus and cortex of sporadic AD and familial AD patients correspond to Aβ_1−42_, Aβ_1−40_, Aβ_4−42_, and the pyroglutamate modified Aβ_3−42_ (Aβ_pE3−42_). It has been demonstrated that N-terminal deletions enhance Aβ aggregation and neurotoxicity *in vitro* when compared to full-length Aβ_1−42_ suggesting that such peptides may initiate and/or accelerate the pathological deposition of Aβ into plaques (Pike et al., 1995). Regardless of the C-terminus of Aβ (Aβ40 or Aβ42), pyroglutaminylated isoforms at position 3 revealed an increased neurotoxicity and higher resistance to degradation than full-length Aβ peptides (Russo et al., 2002; Schlenzig et al., 2009). In accordance with these observations, we have observed that Aβ_pE3−42_ and Aβ_4−42_ are rapidly converted into soluble toxic aggregates *in vitro* and this propensity to form aggregates is more pronounced than N-terminally intact Aβ_1−42_ (Bouter et al., 2013). Transgenic mouse models expressing either Aβ_pE3−42_ or Aβ_4−42_ have been created in order to study the effects of N-truncated Aβ peptides *in vivo* (Wirths et al., 2009; Alexandru et al., 2011; Bouter et al., 2013). The TBA42 mouse model solely expresses Aβ_pE3−42_ (Glu-3 mutated to Gln-3 in order to facilitate pyroGlu-3 formation) and develops intraneuronal Aβ accumulation, massive pyramidal neuron loss in the CA1 region of the hippocampus, motor impairment and behavioral deficits (Wittnam et al., 2012; Meißner et al., 2015). In good agreement with the observations in the TBA42 model, the Tg4-42 mice expressing only intraneuronal Aβ_4−42_ develop severe hippocampal neurons loss accompanied by spatial reference memory deficits (Bouter et al., 2013). However, the Tg4-42 mouse model harbors no mutation in the Aβ sequence. Also, it should be noted that plaque load pathology is low or not observed in these two mouse models. Whether intraneuronal accumulation is part of the AD pathology is unclear. On the other side, it is common knowledge that extracellular Aβ aggregates are of neuronal origin and are secreted as soluble peptides (reviewed in Wirths et al., 2004). Gouras et al. have demonstrated that intraneuronal Aβ may precede tangle formation in the human hippocampus (Gouras et al., 2000). Mochizuki et al. found that Aβ42-positive neurons co-localize with amyloid plaques in AD cases (Mochizuki et al., 2000). Moreover, the observations by Fernandez-Vizarra et al. pointed out that intraneuronal Aβ maybe one of the first neurodegenerative alterations in the AD brain (Fernández-Vizarra et al., 2004). Our current and previously published data demonstrate that both Aβ_pE3−42_ and Aβ_4−42_ play an important role in the pathology of AD. Therefore, the aim of this work was to study a possible effect of Aβ_pE3−42_ and Aβ_4−42_ expression on neuron loss, pathology and neurological deficits in transgenic mice.

## Materials and methods

### Transgenic mice

The generation of TBA42 mice and Tg4-42 has been described previously (Wittnam et al., 2012; Bouter et al., 2013). In brief, TBA42 mice express the murine thyrotropin-releasing hormone-Aβ (mTRH-Aβ_3−42_) under the control of the murine Thy1.2 regulatory sequence. The glutamate at position three of the Aβ amino acid sequence was mutated into glutamine to facilitate enhanced generation of pyroglutamate Aβ_3−42_ (Aβ_pE3−42_) (Wittnam et al., 2012). Tg4-42 mice express the human Aβ_4−42_ sequence fused to the signal peptide sequence of the thyrotropin-releasing hormone under the control of the Thy1 promoter. Bigenic mice were generated by breeding transgene positive TBA42 mice to transgene positive Tg4-42 mice. Wild type and transgenic offspring were identified subsequently using PCR and RT-PCR. All animals were generated and maintained on a C57BL/6J genetic background. Young (2–3 months) and aged (5–6 months) wild type (WT), TBA42 hemizygous (TBA42^hem^), Tg4-42 hemizygous (Tg4-42^hem^), Tg4-42 homozygous (Tg4-42^hom^) and bigenic mice were tested. In the current study, both female and male mice were used. All animals were handled in accordance with the German guidelines for animal care and experiments were approved by the local authorities (Niedersächsisches Landesamt für Verbraucherschutz und Lebensmittelsicherheit, Röverskamp 5, 26203 Oldenburg, Germany; agreement number 15/1760). Due to strong motor deficits, the TBA42 homozygous (TBA42^hom^) mice had to be sacrificed at an age of 2 months. Therefore, these animals could not be used for the behavioral tasks.

### Immunohistochemistry on paraffin sections

Mouse tissue (brain and spinal cord) was processed as described previously (Wirths et al., 2002). In brief, 4-μm paraffin section were deparaffinized in xylene and rehydrated in an ascending series of ethanol baths. After treatment with 0.3% H_2_O_2_ in PBS to block endogenous peroxidases, antigen retrieval was achieved by boiling sections in 0.01 M citrate buffer pH 6.0, followed by 3 min incubation in 88% formic acid. Nonspecific antigens were blocked using a solution of 10% FCS (incl. 4% skim milk) in PBS for 1 h at room temperature (RT) prior to the addition of the primary antibodies. The rabbit polyclonal 24311 (Guzman et al., 2014) (1:500) pan-Aβ antibody (epitope Aβ_4−40_) was incubated overnight in a humid chamber at RT. This was followed by incubation with the corresponding biotinylated secondary antibody (1:200, DAKO, Glostrup, Denmark) at 37°C before visualization of the staining using the ABC method with a Vectastain kit (Vector Laboratories, Burlingame, USA) and diaminobenzidine (DAB) as chromogen. Counterstaining was carried out with hematoxylin.

### Quantification of Aβ deposition

Extracellular and intracellular Aβ deposition was evaluated in the spinal cord. Serial images of 400x magnification were captured on three sections per mouse which were at least 30 μm apart from each other. Slides were imaged using an Olympus BX51 microscope equipped with MoticamPro 282B digital camera. Illumination conditions and exposure settings were kept stable throughout the analysis. Using the Image J software package (V1.41, NIH, USA) the pictures were binarized to 8-bit black and white images and a fixed intensity threshold was applied defining the DAB signal. The percentage of positive DAB staining was calculated as the Aβ deposition load.

### Quantification of motor neurons with low, intermediate, and high intracellular Aβ accumulation

To quantify the number of motor neurons with Aβ accumulation, paraffin-sections of the cervical spinal cord were stained for Aβ (24311, 1:500). Aβ immunopositive motor neurons in the gray matter at the ventral horn were identified by their large size (nuclear diameter >9–10 μ m; cell body diameter >20 μ m). The classification of motor neurons with low, intermediate and high intracellular Aβ accumulation was based on Aβ staining intensity. Serial images of 100x magnification were captured on three sections per animal which were at least 30 μ m apart from each other. For quantification of total Aβ immunopositive motor neurons, the meander scan option of the StereoInvestigator 7 software package (Microbrightfield, Williston, VT, USA) was used.

### Quantification of neuron numbers using design-based stereology

For the stereological analysis, mice were anesthetized and transcardially perfused with 4% paraformaldehyde as previously described (Christensen et al., 2008). In brief, the brain was completely removed from the skull and the left brain hemisphere was postfixed in 4% paraformaldehyde for 2 h, cryoprotected in 30% sucrose, quickly frozen and cut frontally into series of 30 μm thick sections using a cryostat (Leica CM1850 UV, Germany). Every 10th section was systematically sampled, stained with cresyl violet and used for the stereological analysis of the neuron number in the CA1. The CA1 region was counted (Bregma −1.22 to −3.80 mm) using a stereology working station (Olympus BX51 with a motorized specimen stage for automatic sampling, StereoInvestigator 7; Microbrightfield, Williston, USA) and a 100x oil lens (NA = 1.35), neuronal nuclei were sampled uniformly random using optical dissector probes, and the total number of neurons was subsequently estimated by the fractionator method using a 2 μm top guard zone (West et al., 1991).

## Behavioral tasks

### String suspension

The string suspension test was performed to evaluate strength and motor coordination and was described in detail previously (Jawhar et al., 2012). In brief, mice were placed in the middle of an elevated string and permitted to grasp it with their forepaws. For data evaluation, a scoring system of 0–5 was used during a 60 s single trial: 0 = unable to stay on the string; 1 = hanging on the string only by fore- or hind paws; 2 = as for 1, but with attempt to climb onto string; 3 = sits on string and holds balance; 4 = four paws and tail around string with lateral movement; and 5 = escape to one of the platforms.

### Balance beam

The balance beam task was used to assess balance and fine motor coordination (Wirths et al., 2008). Mice were positioned on the center of a 50 cm long and 1 cm wide wooden bar, which is attached to two support columns 44 above a padded surface. At both ends of the bar, 9 cm × 15 wooden escape platforms were installed. Each mouse was given three 60 s trials during a single day of testing. The time each animal remained on the beam was recorded and the resulting time of all three trials was averaged. If an animal remained on the beam for 60 s or escaped to one of the platforms, the maximum time of 60 s was recorded.

### Inverted grip task

Neuromuscular abilities, vestibular function and muscle strength, were tested with the inverted grip task (Wirths et al., 2008). The testing apparatus consisted of a wire grid 45 cm long and 30 cm wide with a grid spacing of 1 cm. The grid was suspended 40 cm above a padded surface using foam supports. Mice were place onto the center of the grid. The grid was inverted and the latency to fall was recorded during a single 60 s trial. If the mice were able to remain on the grid for the entire trial or escaped over the edge of the grid, the maximum time of 60 s was recorded.

### Elevated plus maze

The elevated plus maze test was used to assess anxiety-related behavior (Jawhar et al., 2012). The apparatus consisted of four arms raised 75 cm above the floor with two 15 cm long and 5 cm wide open and enclosed arms with 15 cm high walls. The mouse was positioned in the 25 cm^2^ central region of the maze facing one of the open arms and allowed to freely explore the maze during a single 5 min trial. The percentage of the time spent in the open arms and the distance traveled was measured using an automatic video tracking system (ANY-maze, Stoelting, USA).

### Statistical analysis

Differences between groups were tested with one-way analysis of variance (ANOVA) followed by Bonferroni's *post-hoc* or unpaired *t*-test, as indicated. All data were given as means ± standard error of the mean (SEM). Significance levels were given as follows: ^***^p < 0.001; ^**^p < 0.01; ^*^p < 0.05. All calculations were performed using GraphPad Prism version 7 for Windows (Graph Pad Software, San Diego, USA).

## Results

### Strong Aβ accumulation in the CA1 region of the hippocampus in bigenic mice

**Figure 1:**
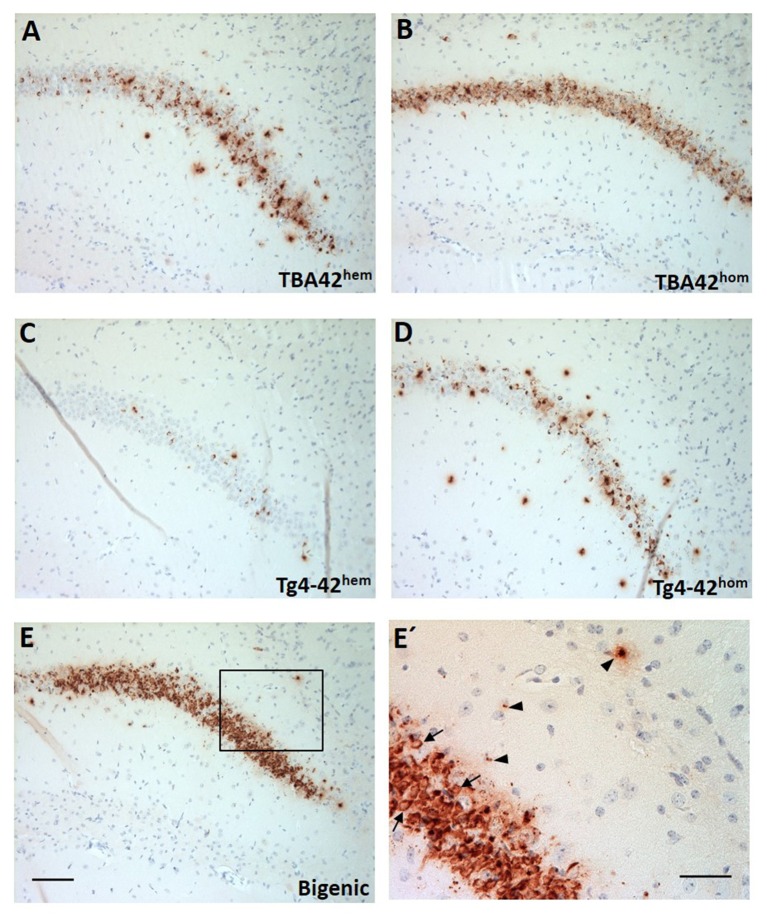
Strong Aβ intraneuronal accumulation in the CA1 pyramidal cell layer of the hippocampus in the bigenic mice. Immunohistochemistry using a pan-Aβ antibody (24311) showed immunoreactivity already in young mice **(A–E)**. Particularly, abundant intraneuronal Aβ accumulation could be observed in young TBA42^hom^ **(B)** and bigenic mice **(E)**. **(E')** Represents a magnification of **(E)** showing intraneuronal Aβ accumulation (arrows) and extracellular Aβ deposition (arrowheads). Scale bars, **A–E** = 100 μm and **E** = 50 μm.

Brain sections of TBA42^hem^, TBA42^hom^, Tg4-42^hem^, Tg4-42^hom^, and bigenic mice were immunostained with the pan-Aβ antibody 24311 to assess the expression of Aβ_pE3−42_ and Aβ_4−42_. All young mice showed Aβ intraneuronal accumulation in the CA1 pyramidal cell layer of the hippocampus (Figures 1A–E), particularly abundant intraneuronal Aβ immunoreactivity could be seen in TBA42^hom^ (Figure 1B) and bigenic mice (Figures 1E,E'). Aβ immunoreactivity in the CA1 region declined with age in all analyzed genotypes (data not shown).

### Accelerated neuron loss in the hippocampus of bigenic mice

**Figure 2:**
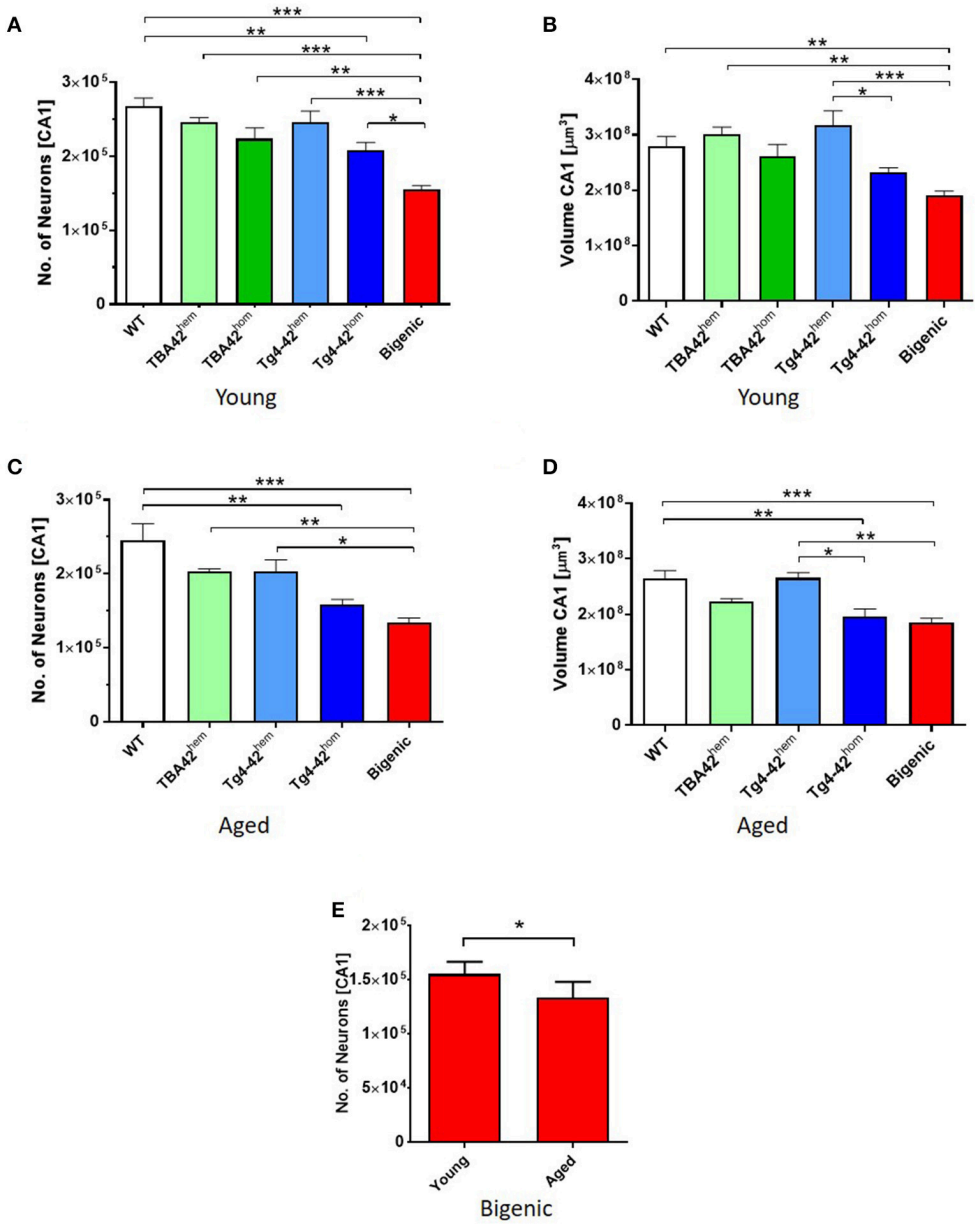
Enhanced neuron loss in the CA1 pyramidal cell layer of the hippocampus in bigenic mice. Design-based stereological analysis revealed a significantly reduced CA1 neurons numbers in young (2–3 months) bigenic mice when compared to the rest of the age-matched groups **(A)**. Reduction in the CA1 volume of young bigenic mice could be detected when compared to same age WT, TBA42^hem^ and Tg4-42^hem^. No differences in volume were found between young TBA42^hom^, Tg4-42^hom^, and bigenic mice **(B)**. Neuron loss continued in aged (5–6 months) bigenic mice **(C)**. Aged bigenic mice showed reduced CA1 volume when compared to the rest of the age-matched groups with exception of TBA42^hem^ and Tg4-42^hom^ mice **(D)**. No differences in the CA1 neuron numbers between aged Tg4-42^hom^ and bigenic mice were detected. **(E)** Neuron numbers are significantly decreased in bigenic mice in an age-dependent manner. **(A–D)** One-way ANOVA followed by Bonferroni's *post-hoc* test. **(E)** Unpaired *t*-test. All data were given as means ± SEM ^*^p < 0.05; ^**^p < 0.01; ^***^p < 0.001; *n* = 3–5 per group.

To analyze the impact of the co-expression of Aβ_pE3−42_ and Aβ_4−42_ on CA1 neuron numbers of bigenic mice, unbiased design-based stereology measurements were performed. Young bigenic mice showed a 41% neuron loss (Figure 2A; *p* < 0.001, 155,389 ± 5,103) in the CA1 region of the hippocampus compared to WT mice (267,767 ± 11,196). In young TBA42^hem^ (246,145 ± 6,280), TBA42^hom^ (223,937 ± 14,553), and Tg4-42^hem^ (245,925 ± 15,234) mice no significant neuron loss could be detected when compared to age-matched WT mice, while Tg4-42^hom^ mice showed already a 20% neuron loss compared to WT (*p* < 0.01, 208,057 ± 10,452). Likewise, young bigenic mice showed reduced neuron numbers relative to TBA42^hem^ (*p* < 0.001), TBA42^hom^ (*p* < 0.01), Tg4-42^hem^ (*p* < 0.001), and Tg4-42^hom^ mice (*p* < 0.05). In aged TBA42^hem^ (203,465 ± 3,140) and Tg4-42^hem^ (203,092 ± 15,743) mice, no significant neuron loss was observed when compared to age-matched WT controls (244,941 ± 22,750) (Figure 2C). Bigenic mice (133,707 ± 6,494) revealed reduced neuron numbers in comparison to WT (*p* < 0.001), TBA42^hem^ (*p* < 0.01) and Tg4-42^hem^ (*p* < 0.05) mice. No significant differences could be observed in the number of neurons in the CA1 pyramidal cell layer between Tg4-42^hom^ (158,246 ± 7,186) and bigenic mice. A quantitative analysis of the CA1 volume demonstrated a significant reduction of ~31% in the young bigenic mice in relation to WT controls (*p* < 0.01; Figure 2B). Moreover, young bigenic mice displayed a reduced CA1 volume compared to TBA42^hem^ and Tg4-42^hem^, whereas no differences in CA1 volume were found between young bigenic, TBA42^hom^ and Tg4-42^hom^ animals. The reduction in CA1 volume was preserved in aged bigenic mice, as seen by a decreased volume when compared to WT (*p* < 0.001) and Tg4-42^hem^ (*p* < 0.01) mice of the same age (Figure 2D). At this time point, Tg4-42^hom^ also showed significant differences compared to age-matched WT (*p* < 0.01) and Tg4-42^hem^ (*p* < 0.05) mice, while no differences in the CA1 volume between aged bigenic and Tg4-42^hom^ mice could be detected. Neuron loss increased in bigenic mice in an age-dependent manner (unpaired *t*-test, *p* < 0.05; Figure 2E).

### Amyloid pathology in the spinal cord of bigenic mice

**Figure 3:**
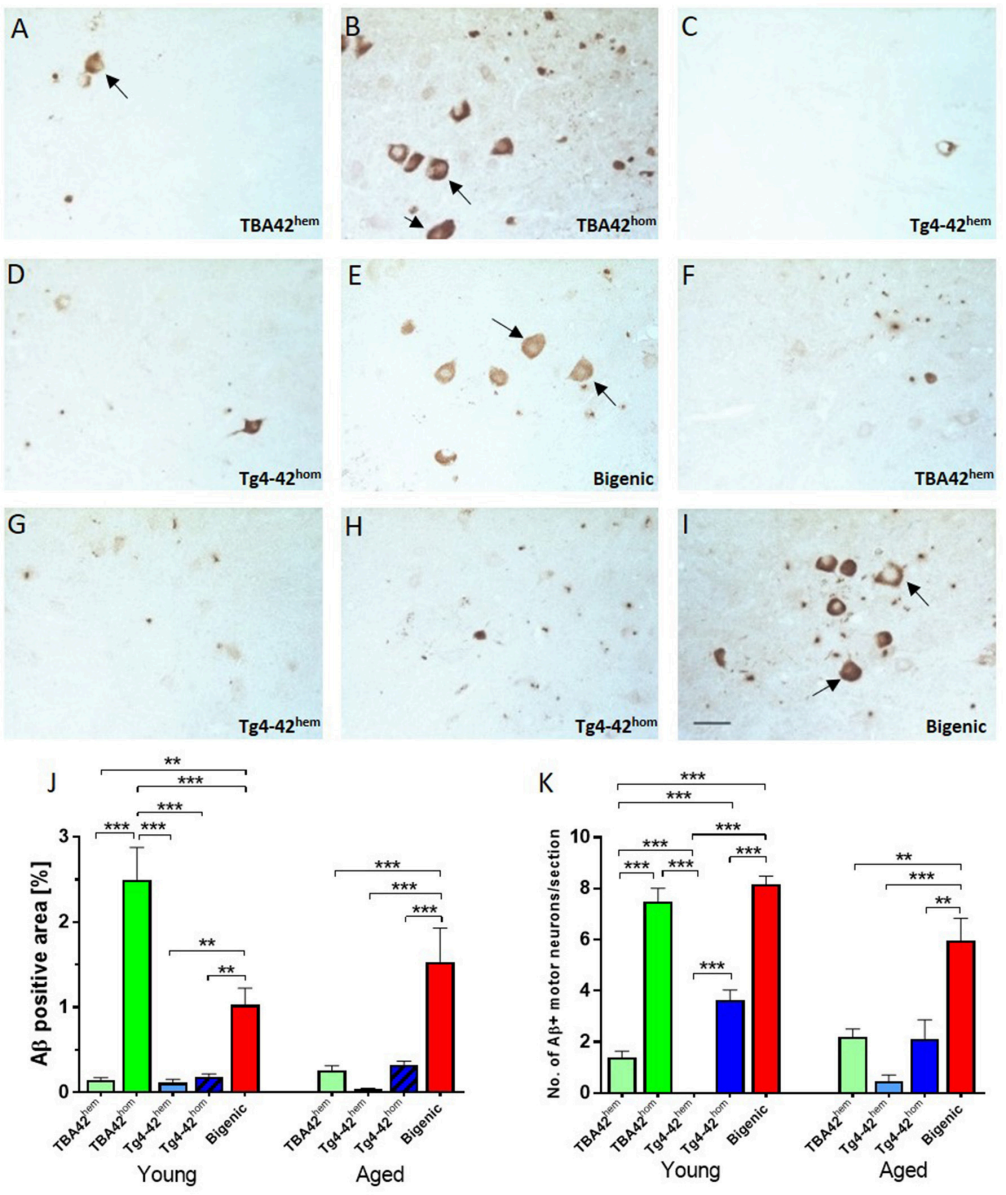
Extra and intraneuronal Aβ deposition in the spinal cord of transgenic mice. Staining against Aβ revealed extra and intraneuronal accumulation in the motor neurons (arrows) of the spinal cord starting at young age **(A–E)**, which increased in aged mice **(F–I)**. Quantification of Aβ deposition showed a high amyloid pathology already in young TBA42^hom^ and bigenic mice which was exacerbated in aged bigenic mice **(J)**. Similarly, the total number of Aβ-positive motor neurons was significantly higher in young TBA42^hom^ and bigenic mice when compared to the rest of the groups. A higher number Aβ-positive motor neurons was also observed in aged bigenic mice **(K)**. One-way ANOVA followed by Bonferroni's *post-hoc* test. All data were given as means ± SEM ^**^p < 0.01; ^***^p < 0.001; *n* = 3–5 per group; scale bar = 50 μm.

Aβ_pE3−42_ and Aβ_4−42_ are expressed under the control of the neuron-specific murine Thy-1 promoter, which is active in both hippocampus and spinal cord. Immunohistochemical analysis of spinal cord sections showed Aβ deposition already in young single transgenic and bigenic mice (Figures 3A–I). Quantification of extra- and intraneuronal Aβ revealed significant differences between young TBA42^hom^ mice and the other age-matched groups analyzed. Additionally, young bigenic mice exhibited an increased amyloid pathology when compared to young TBA42^hem^ (*p* < 0.01), Tg4-42^hem^ (*p* < 0.01), and Tg4-42^hom^ (*p* < 0.01) mice (Figure 3J). The same held true for aged bigenic mice where amyloid pathology was increased in comparison to age-matched TBA42^hem^ (*p* < 0.001), Tg4-42^hem^ (*p* < 0.001), and Tg4-42^hom^ (*p* < 0.001) mice (**Figure 5J**). Weak Aβ immunoreactivity was detected in the spinal cord of TBA42^hem^, Tg4-42^hem^, and Tg4-42^hom^ at all time points analyzed.

### High Aβ accumulation in the motor neurons of bigenic mice

Immunostaining of spinal cord sections of single and double transgenic mice using a pan-Aβ antibody revealed intracellular Aβ accumulation in motor neurons of the ventral horn. Quantification of the total Aβ immunopositive motor neurons revealed higher numbers in young bigenic mice than TBA42^hem^ (*p* < 0.001), Tg4-42^hem^ (*p* < 0.001), and Tg4-42^hom^ (*p* < 0.001) mice. Similarly, young TBA42^hom^ mice showed higher numbers when compared to same age TBA42^hem^ (*p* < 0.001) and Tg4-42^hem^ (*p* < 0.001) mice (Figure 3K). Young Tg4-42^hom^ mice revealed higher Aβ immunopositive motor neurons than TBA42^hem^ (*p* < 0.001) and Tg4-42^hem^ (*p* < 0.001). Significant differences were found between aged bigenic mice and TBA42^hem^ (*p* < 0.01), Tg4-42^hem^ (*p* < 0.001), and Tg4-42^hom^ (*p* < 0.01) mice.

**Figure 4:**
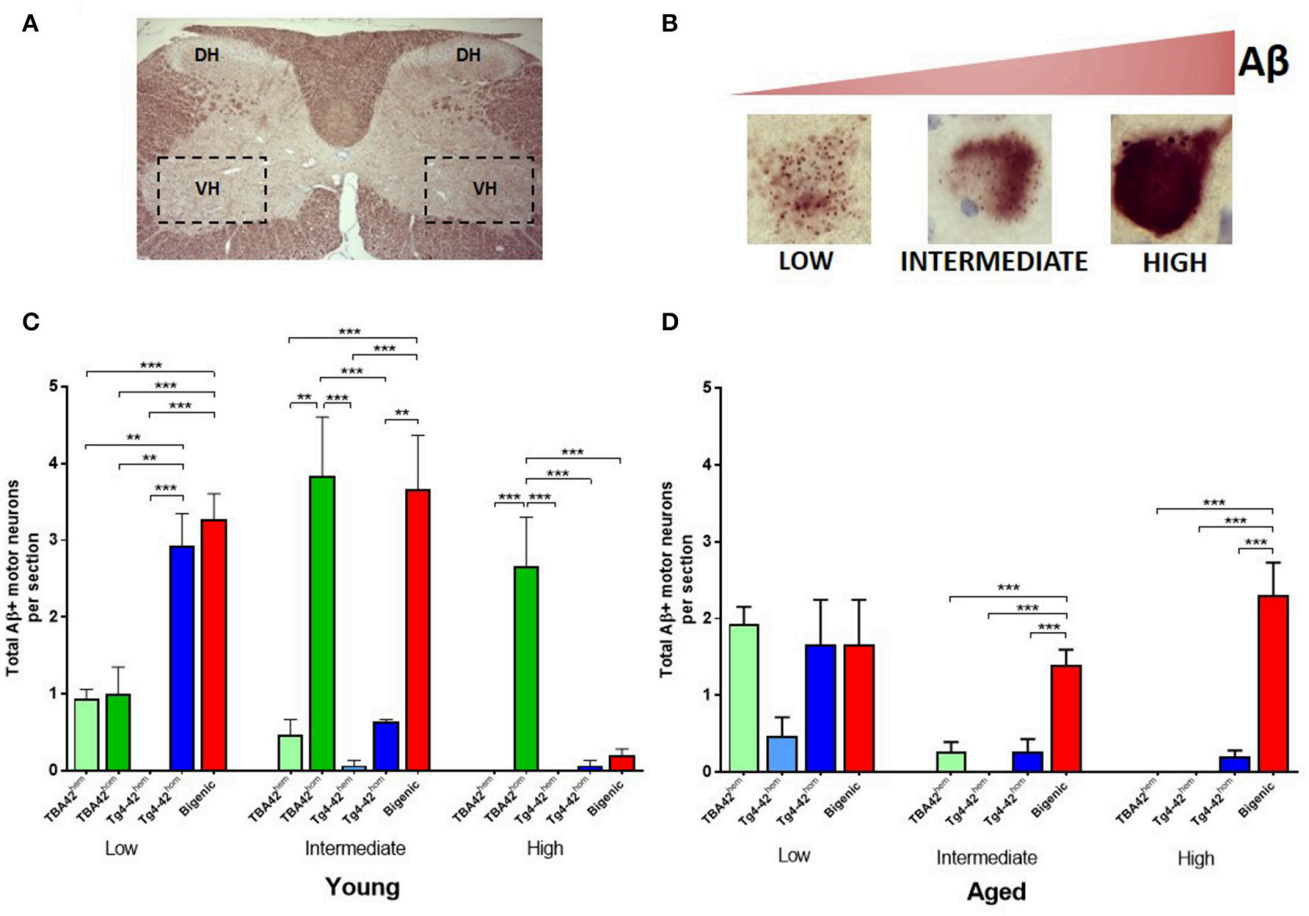
Quantification of intracellular Aβ accumulation in motor neurons of transgenic mice with varying levels of Aβ. Schematic picture of the cervical region of the spinal cord **(A)** showing the dorsal horn (DH) and the ventral horn (VH). Three different Aβ intraneuronal accumulation levels could be found in the motor neurons of VH and were defined as: low, intermediate and high **(B)**. Quantitative analysis of the Aβ intraneuronal levels in the spinal cord young mice revealed a higher number of Aβ positive motor neurons with low Aβ accumulation in Tg4-42^hom^ and bigenic mice. Intermediate accumulation was similar in TBA42^hom^ and bigenic mice and high accumulation was found only in TBA42^hom^ mice **(C)**. In aged mice, motor neuron numbers with low Aβ accumulation were similar in all groups. However, more motor neurons showed intermediate and high Aβ levels in the aged bigenic mice **(D)**. One-way ANOVA followed by Bonferroni's *post-hoc* test. All data were given as means ± SEM ^**^p < 0.01; ^***^p < 0.001; *n* = 3–5 per group.

Quantitative analysis of the total number of motor neurons with low, intermediate and high intracellular Aβ accumulation was performed in young and aged mice (Figures 4A–D). The results revealed a higher number of motor neurons with low Aβ accumulation in young Tg4-42^hom^ and bigenic mice, compared to age-matched TBA42^hem^, TBA42^hom^, and Tg4-42^hem^ animals. Intermediate accumulation in young mice was similar in the TBA42^hom^ and bigenic groups, whereas motor neurons with high intracellular Aβ accumulation were only found in TBA42^hom^ mice (Figure 4C). In aged mice, no differences in the total number of motor neuron with low Aβ accumulation were found in any of the groups analyzed. Nevertheless, increased numbers of motor neurons with intermediate and high intracellular Aβ levels were found only in the bigenic mice (Figure 4D).

### Reduced anxiety levels in the bigenic mice

**Figure 5:**
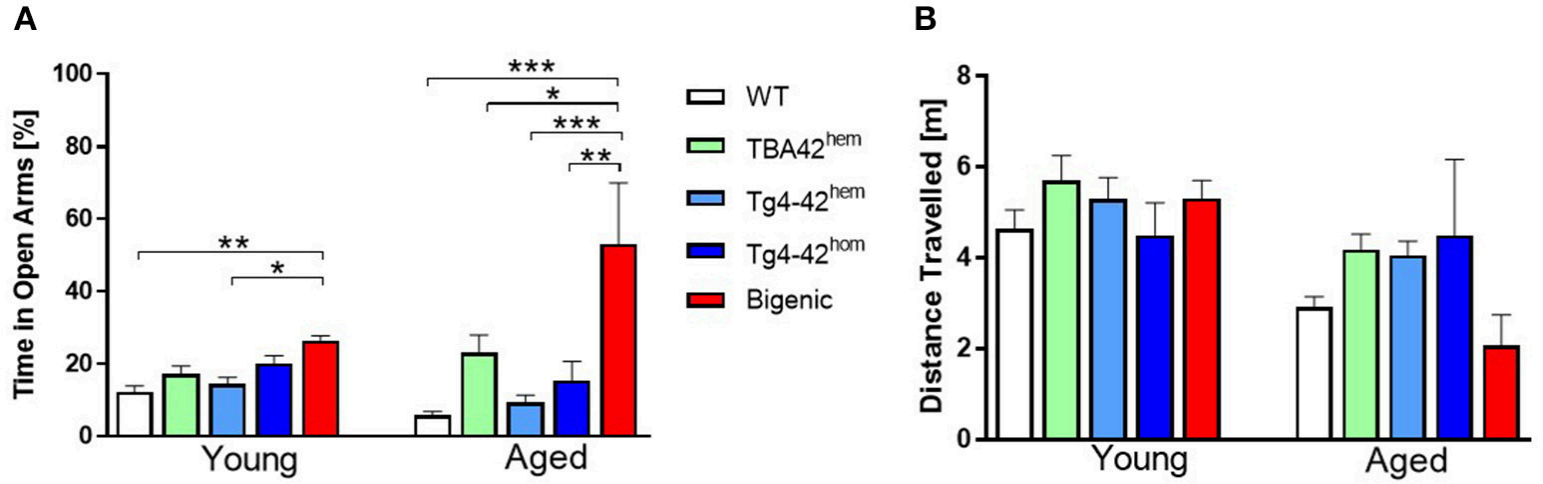
Reduced anxiety levels in bigenic mice. Reduced anxiety could be observed already in young bigenic mice as reflected by significantly greater amount of time spent in the open arms when compared to WT and Tg4-42^hem^ mice. Anxiety levels increased in an age-dependent manner in bigenic mice **(A)**. No difference in the distance traveled in the young or aged mice could be detected **(B)**. One-way ANOVA followed by Bonferroni's *post-hoc* test. All data were given as means ± SEM ^*^p < 0.05; ^**^p < 0.01; ^***^p < 0.001; *n* = 4–12.

The elevated plus maze test was used to study anxiety levels in young and aged TBA42, Tg4-42 and bigenic mice. Young bigenic mice showed reduced anxiety levels compared to WT (*p* < 0.01) and Tg4-42^hem^ (*p* < 0.05) mice (Figure 5A), shown by a higher percentage of time spent in the open arms. No change in the anxiety-like behavior was found in young TBA42^hem^, Tg4-42^hem^, and Tg4-42^hom^. The anxiety levels were even further decreased in aged bigenic mice when compared to age-matched WT (*p* < 0.001), TBA42^hem^ (*p* < 0.05), Tg4-42^hem^ (*p* < 0.001), and Tg4-42^hom^ (*p* < 0.01). In addition, the distance traveled was used as an index of general activity and did not differ between all groups analyzed (Figure 5B).

### Co-expression of Aβ_pE3−42_ and Aβ_4−42_ aggravates sensori-motor function in an age-dependent manner

**Figure 6:**
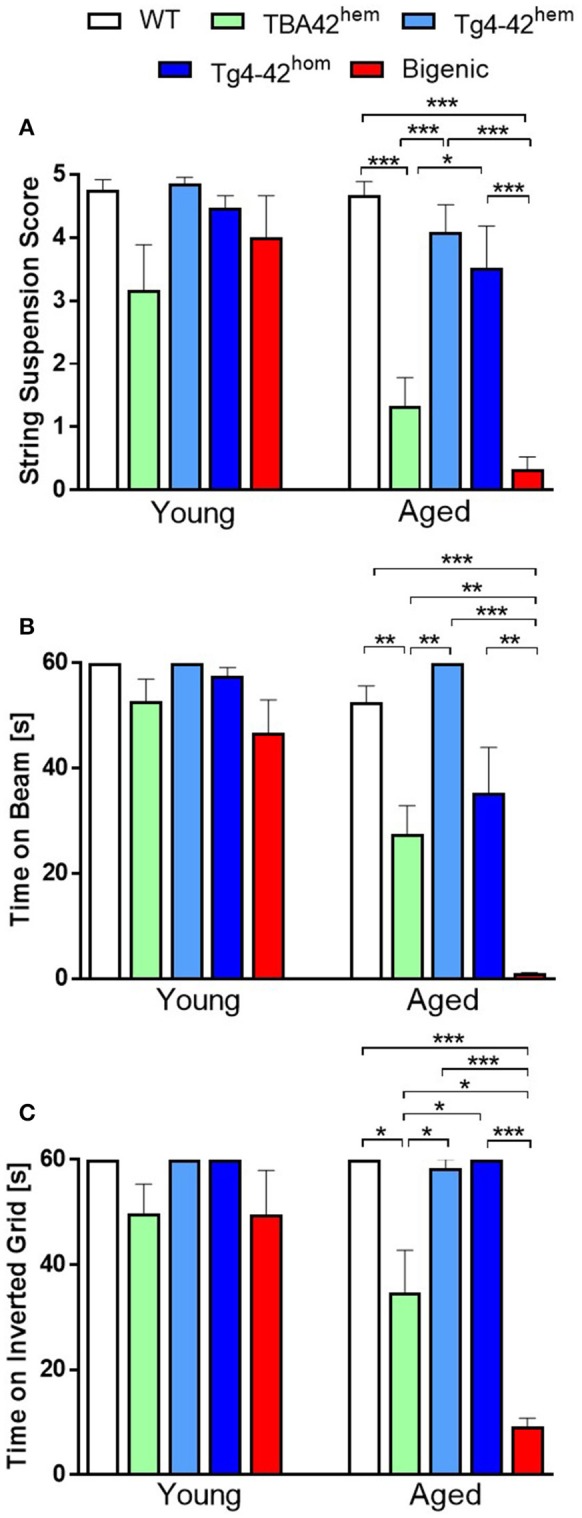
Severe sensori-motor deficits in bigenic mice. The string suspension **(A)**, the balance beam **(B)** and the inverted grid task **(C)** revealed severe sensori-motor deficits in bigenic mice in an age-dependent manner in comparison to the rest of the groups analyzed. Yet, no significant difference could be observed between aged TBA42^hem^ and bigenic mice in the string suspension task. One-way ANOVA followed by Bonferroni's *post-hoc* test. All data were given as means ± SEM ^*^p < 0.05; ^**^p < 0.01; ^***^p < 0.001; *n* = 5–12 per group.

To evaluate the effect of the combination of Aβ_pE3−42_ and Aβ_4−42_ on sensory-motor abilities in the bigenic mice, the string suspension, the balance beam and the inverted grip tasks were carried out (Figure 6). Performance in all three tests declined in bigenic mice in age-dependent manner. The string suspension task evaluates sensori-motor strength and coordination by measuring the ability of mice to remain on a string. No difference in the scores was observed in young transgenic mice compared to WT (Figure 6A). However, aged bigenic mice showed a poorer performance compared to WT, Tg4-42^hem^ and Tg4-42^hom^ (*p* < 0.001 in all groups). Aged TBA42^hem^ also performed poorly compared to WT (*p* < 0.001), Tg4-42^hem^ (*p* < 0.001) and Tg4-42^hom^ (*p* < 0.05). No significant difference could be observed between TBA42^hem^ and bigenic mice in this task. The balance beam task was used to assess balance and fine motor coordination. No impairment in this task was detected in young mice (Figure 6B), while aged bigenic mice performed worse than age-matched WT (*p* < 0.001), TBA42^hem^ (*p* < 0.01), Tg4-42^hem^ (*p* < 0.001), and Tg4-42^hom^ (*p* < 0.01). Sensori-motor deficits were also observed in aged TBA42^hem^ mice when compared WT (*p* < 0.01) and Tg4-42^hem^ (*p* < 0.01). Sensori-motor abilities, vestibular function and muscle strength were tested with the inverted grip task by analyzing the latency to fall (Figure 6C). As seen in the other tasks, no motor deficits could be detected in young bigenic mice. Nevertheless, aged bigenic mice demonstrated strong sensori-motor deficits shown by shorter latencies to fall compared to same age WT (*p* < 0.001), TBA42^hem^ (*p* < 0.05), Tg4-42^hem^ (*p* < 0.001), and Tg4-42^hom^ (*p* < 0.001). Likewise, aged TBA42^hem^ performed worse than WT (*p* < 0.05), and Tg4-42^hem^ (*p* < 0.05).

Altogether, these results revealed that the combined expression of Aβ_pE3−42_ and Aβ_4−42_ exacerbates the sensori-motor deficits already seen in the TBA42 mouse model, which uniquely accumulates Aβ_pE3−42_.

## Discussion

Already three decades ago, it was discovered that more than 60% of the Aβ peptides purified from the amyloid plaque cores of AD brains started with Phenylalanine at position 4 of the Aβ sequence (Masters et al., 1985). Further studies corroborated these results and demonstrated that in addition to Aβ_4−42_ and the full-length Aβ_1−42_ isoform, Aβ_pE3−42_ is highly abundant in AD brains (Masters et al., 1985; Saido et al., 1995; Portelius et al., 2010). Studies trying to unravel the pathogenic properties of these two N-truncated species have been performed in recent years. Pike et al. reported that N-terminal truncations enhance peptide aggregation and neurotoxicity in relation with full-length Aβ (Pike et al., 1995). They compared the biophysical and bioactive properties of Aβ peptides starting at positions Aspartate-1, Phenylalanine-4, Serine-8, Valine-12, and Lysine-17 with C-termini extending to residue 40 or 42. Overall, peptides with N-terminal deletions and terminating at residue 42 exhibited enhanced peptide aggregation relative to full-length species. In addition, N-truncated peptides showed fibrillar morphology under transmission electron microscopy, and significant toxicity in cultures of rat hippocampal neurons. Furthermore, Russo and colleagues reported that pyroglutamate-modified Aβ peptides starting at position 3 (Aβ_pE3−40/42_) are more toxic than full-length Aβ (Russo et al., 2002). Additionally, they found that fiber morphology is greatly influenced by the C-terminus whilst cellular toxicity and degradation are influenced by the N-terminus. Our previously published data extended these observations. We have demonstrated that soluble aggregates of Aβ_4−42_ and Aβ_pE3−42_ have specific features that might carry their neurotoxic activity (Bouter et al., 2013). These soluble aggregates were capable of converting to fibrillar aggregates as shown by a high Thioflavin-T-reactivity already during the nucleation phase of aggregation. We also demonstrated by using far-UV CD spectroscopy, NMR spectroscopy and dynamic light scattering that Aβ_4−42_ and Aβ_pE3−42_, and to a lesser extent Aβ_1−42_, had a remarkable tendency to form stable aggregates. Furthermore, we observed that short-term exposure of Aβ_4−42_ peptides have a cytotoxic effect in primary cortical cultures, demonstrating that Aβ_4−42_ can be as toxic as Aβ_1−42_ and Aβ_pE3−42_ (Bouter et al., 2013)_._

In order to study the direct *in vivo* toxicity of Aβ_pE3−42_ and Aβ_4−42_, transgenic mouse models expressing uniquely the respective N-truncated Aβ peptides have been developed. The TBA42 mouse model, expresses Aβ starting with an N-terminal glutamine residue at position three, which has been demonstrated to represent a better substrate for both the spontaneous and enzymatic conversion of Aβ_3−42_ into Aβ_pE3−42_ (Wittnam et al., 2012). Phenotypical characterization of this mouse model showed that TBA42 mice exhibit an accumulation of intraneuronal Aβ in CA1 pyramidal neurons, followed by significant neuron loss, behavioral and motor deficits that increased in an age-dependent manner (Meißner et al., 2015). Similarly, other mouse models expressing uniquely Aβ_pE3−42_ have demonstrated the *in vivo* toxicity of this peptide (Wirths et al., 2009; Alexandru et al., 2011). However, the degree of conversion was not determined for these models. Hence, it cannot be excluded that the unmodified Aβ_3−42_ may contribute to the pathological and behavioral deficits observed in these mice. On the other hand, in order to investigate the *in vivo* toxic effects of Aβ_4−42_ only, we have previously developed a transgenic mouse expressing human Aβ_4−42_ without any mutation (Tg4-42 mouse model). These mice are characterized by a robust age-dependent neuron loss in the CA1 pyramidal layer, which coincides with the intraneuronal Aβ accumulation observed in the hippocampus (Bouter et al., 2013). Furthermore, these mice developed age-dependent behavioral deficits. In sum, it is clear that both, Aβ_pE3−42_ and Aβ_4−42_ have a neurotoxic *in vivo* effect when individually expressed in transgenic mice.

Therefore, the aim of the present work was to study the expressing both N-truncated Aβ peptides and try to elucidate a possible effect on neuron loss, neuropathology and neurological deficits in transgenic mice. Our observations suggest that Aβ_pE3−42_ and Aβ_4−42_ enhance their toxicity when combined, resulting in an accelerated neuronal death.

The gene-dosage does have an effect on the outcome of the current work. In fact, we have shown that homozygous TBA42 or homozygous Tg4-42 mice develop enhanced neuron loss in the CA1 area and neuropathological alterations at the same age as compared to hemizygous TBA42 or hemizygous Tg4-42 mice. This clearly demonstrates a gene-dosage effect. The effect on neuron loss, neuropathological alterations and neurological deficits in bigenic mice was however stronger compared to homozygous TBA42 or homozygous Tg4-42 mice. It is important to note that homozygous TBA42, homozygous Tg4-42 and bigenic mice express the same level of Aβ with a distinct difference: homozygous TBA42 mice express only pyroglutamate Aβ3-42, homozygous Tg4-42 mice express only Aβ4-42, whereas bigenic mice express both peptides together.

Neuron loss has been reported in other AD mouse models (Casas et al., 2004; Oakley et al., 2006; Breyhan et al., 2009; Christensen et al., 2010). Interestingly, the neuron loss observed in these animals occurs in the brain regions with robust intraneuronal Aβ accumulation. In line with these observations, our current results support the role of intraneuronal Aβ as a trigger of the pathological events leading to neurodegeneration in AD (Wirths et al., 2004). Likewise, a reduction of the anxiety levels in the bigenic animals could be detected at an early age, which further increased in an age-dependent manner. This hypo-anxious phenotype could be a consequence of the massive neuron loss observed in the CA1 region of the hippocampus of bigenic mice. However, a possible altered function in other circuitries of the limbic system should not be ruled out (Lalonde et al., 2012). Besides the neurobehavioral and neuropsychiatric symptoms observed typically in AD patients (Chung and Cummings, 2000), motor impairments, including rigidity and disturbances in gait or posture have also been repeatedly reported (O'Keeffe et al., 1996; Kluger et al., 1997; Scarmeas et al., 2004; Pettersson et al., 2005). Very recently, an association of brain amyloid-β, assessed by cerebral Pittsburgh Compound B (PiB) positron emission tomography, and slower gait was also reported in elderly individuals without dementia (Nadkarni et al., 2017). This suggests that motor impairment is an important aspect of cognitive decline in AD. Alteration of motor abilities have been also demonstrated in different AD mouse models (Lalonde et al., 2002, 2004; Wirths and Bayer, 2008; Wirths et al., 2008; Jawhar et al., 2012; Meißner et al., 2015). Here, we report that mice co-expressing Aβ_pE3−42_ and Aβ_4−42_ exhibit severe motor deficits. However, it should be noted that the presence of Aβ_pE3−42_ is crucial for the observed sensori-motor phenotype in the bigenic mice, since no sensori-motor deficiencies could be observed in either Tg4-42 hemi- or homozygous mice. Moreover, the sensori-motor deficits seen in the bigenic mice nicely correlate with the significant extra- and intraneuronal Aβ deposition observed in the spinal cord of these animals. Yet, significant amyloid pathology was also found in young TBA42^hom^ mice, suggesting that Aβ_pE3−42_ might co-aggregate with Aβ_4−42_. The results of the present study demonstrate the toxicity of N-truncated Aβ peptides in transgenic mice. The transgenic models therefore only partially mimic AD-typical pathology like CA1 neuron loss. In the clinical form of AD neurofibrillary tangles and extracellular amyloid plaques are major hallmarks of the pathology, which are absent in the models studied here. A limitation of the study is that we could not analyze the hippocampus-related spatial reference memory in the Morris water maze test due to the sensori-motor deficits of the bigenic mice. The spinal pathology and associated sensori-motor deficits are likely due to the Thy1 promotor of the transgenic expression vectors and do not model Alzheimer pathology in humans. Nevertheless, the alterations in the spinal cord model the toxic effects of N-truncated Aβ peptides in mouse brain, which is interesting as such. Unfortunately, we could not study the amyloid pathology in aged TBA42^hom^ due to the severe sensori-motor deficits observed in these animals already at a young age. In a previous study, we could corroborate the relevance of Aβ_pE3−42_ in the etiology of AD. To this end, we crossed 5XFAD mice with TBA42 mice. The resulting transgenic mice (FAD42), showed a significant increase in the ratio of Aβ_pE3_ to Aβ_1−x_ cortical plaque load between 5XFAD and FAD42. This data coincides with the enhanced behavioral deficits observed in the FAD42 in relation to 5XFAD and TBA42 mice (Wittnam et al., 2012).

Several studies have demonstrated that N-terminal pyroglutamate modification of Aβ increases its toxicity (Schlenzig et al., 2009, 2012), resistance to degradation by aminopeptidases (Saido et al., 1995), as well as the aggregation kinetics (He and Barrow, 1999; Schilling et al., 2006; Schlenzig et al., 2009). Schilling et al. have demonstrated that pyroglutamate Aβ peptides exhibit biophysical characteristics that might be in particular crucial for the initiation of the disease (Schilling et al., 2006). Mixed aggregates consisting of either pyroglutamate Aβ_3−42_ and full-length Aβ_1−42_ show enhanced aggregation suggesting that pyroglutamate Aβ_3−42_ (Schilling et al., 2006, 2008). Nussbaum et al. corroborated these results by demonstrating that small amounts of pyroglutamate Aβ_3−42_ co-oligomerized with excess of full-length Aβ_1−42_ *in vitro*, thereby potentiating the toxicity of Aβ_1−42_ by inducing the formation of toxic mixed oligomers (Nussbaum et al., 2012). Pyroglutamate Aβ_3−42_ induced tau-dependent neuronal death and template-induced misfolding of Aβ_1−42_ into structurally distinct low-molecular weight oligomers that propagated by a prion-like mechanism (Nussbaum et al., 2012).

More recently, Dammers et al. elucidated the co-aggregation mechanism of Aβ_pE3−42_ with Aβ_1−42_ (Dammers et al., 2017). They found that Aβ_pE3−42_ monomers increase the primary nucleation of Aβ_1−42_ and Aβ_pE3−42_ fibrils are efficient templates for Aβ_1−42_ elongation. Interestingly, fibrils of Aβ_1−42_ prevent Aβ_pE3−42_ aggregation. Thus, it cannot be ruled out that a similar mechanism of co-aggregation between Aβ_pE3−42_ and Aβ_4−42_ may partially explain the observed phenotype in mice co-expressing the two N-truncated peptides. Continuative studies involving the interaction of Aβ_4−42_ with other Aβ isoforms would allow us to better understand the impact of this peptide in AD. In the present study, for the first time, we provided evidence for a possible *in vivo* interaction between Aβ_pE3−42_ and Aβ_4−42_. This seems plausible, as both peptides are two of the most abundant Aβ species found in the brain of AD patients. We expanded our previous studies demonstrating the potential role of N-truncated Aβ peptides in AD pathogenesis. Hence, we suggest that both peptides together, Aβ_pE3−42_ and Aβ_4−42_ are relevant therapeutic targets to fight AD.

## Author contributions

JL-N performed experiments, analyzed data and wrote the manuscript. NG, MU, JM, CP, YB, and JA performed experiments. OW, YB, and TB designed and supervised the work.

### Conflict of interest statement

The Tg-4-42 mouse model has been patented by the University Medicine Göttingen. Inventors are OW and TB. The other authors declare that the research was conducted in the absence of any commercial or financial relationships that could be construed as a potential conflict of interest.
